# Supplementary material for: A New Xanthone Glycoside from Mangifera indica L.: Physicochemical Properties and In Vitro Anti-Skin Aging Activities
Source: Molecules. 2022 Apr 19;27(9):2609. doi: 10.3390/molecules27092609 (PMC9105941; doi:10.3390/molecules27092609)

A New Xanthone Glycoside from *Mangifera indica* L.: Physicochemical Properties and *in vitro* Anti-skin Aging Activities

Heba A.S. El-Nashar[1,2*], Eman M. El-labbad [3,4*], Mahmood A. Al-Azzawi[5], Naglaa S. Ashmawy[1,6]

[1] *Department of Pharmacognosy, Faculty of Pharmacy, Ain Shams University, Abbassia, Cairo, Egypt.*

[2] *Centre of Drug Discovery Research and Development, Ain Shams University, Cairo, Egypt.*

[3] *Department of Pharmaceutical Sciences, College of Pharmacy, Gulf Medical University, Ajman, United Arab Emirates*

[4] *Pharmaceutical Chemistry Department, Faculty of Pharmacy, Ain Shams University, Abbassia, 11566, Cairo, Egypt*

[5] *Department of Pathological Analysis Techniques, College of Medical & Health Technologies, Ahl Al Bayt University, Karbala, Iraq.*

[6] *Rochester Institute of Technology-Dubai, Dubai, UAE.*

*Corresponding authors: Heba A.S. El-Nashar: heba_pharma@pharma.asu.edu.eg , Department of Pharmacognosy, Faculty of Pharmacy, Ain Shams University, 11566 Cairo, Egypt. Tel: +2 02 2405 1120. Fax: +2 02 2405 1107.

Eman M. El-labbad: Dr.eman.m@gmu.ac.ae, Department of Pharmaceutical Sciences, College of Pharmacy, Gulf Medical University, Ajman, United Arab Emirates, P.O - Box : 4184 - Ajman, UAE. Tel:+971 6 7431333

ABSTRACT

A new xanthone glycoside, 1,3,5,6-tetrahydroxyxanthone-C-4-β-D-glucopyranoside was isolated from the methanol extract of Mangifera indica leaves (Anacardiaceae) growing in Egypt. The structure was clarified by 1D and 2D-NMR spectroscopic data. The physicochemical properties of compound like lipophilicity, solubility and formulation considerations were predicted via in silico ADMET technique using SwissADME server. This technique provided Lipinski's rule of five, such as GIT absorption, distribution, metabolism, and skin permeation. The in-vitro inhibitory activities against aging-mediated enzymes like collagenase, elastase, hyaluronidase and tyrosinase were as-sessed. The compound exhibited remarkable anti-collagenase, anti-elastase, anti-hyaluronidase and anti-tyrosinase effects with IC50 values of 1.06, 419.10, 1.65 and 0.48 µg/mL, respectively, compared to the positive control. The compound showed promising predicted aqueous solubility and rea-sonable skin penetration suggesting the suitability of compound for topical formulation as antiaging agent for cosmetic preparations.
Keywords: xanthone, *Mangifera indica*, aging, collagenase, elastase, tyrosinase, hyaluronidase, *in silico* ADMET.

Spectra of compound 1

Figure S1. ^{1}H-NMR spectrum of isolated compound (full spectrum).

Figure S2. ^{1}H-NMR spectrum of isolated compound (aromatic region).

Figure S3. APT spectrum of isolated compound (full spectrum)

Figure S4. HMBC spectrum of isolated compound

Figure S5. HSQC spectrum of isolated compound

Figure S6. ^{1}H,^{1}H-COSY spectrum of isolated compound

Figure S7. (-)ESI-MS of isolated compound

Figure S1. ^{1}H-NMR spectrum of isolated compound (full spectrum).

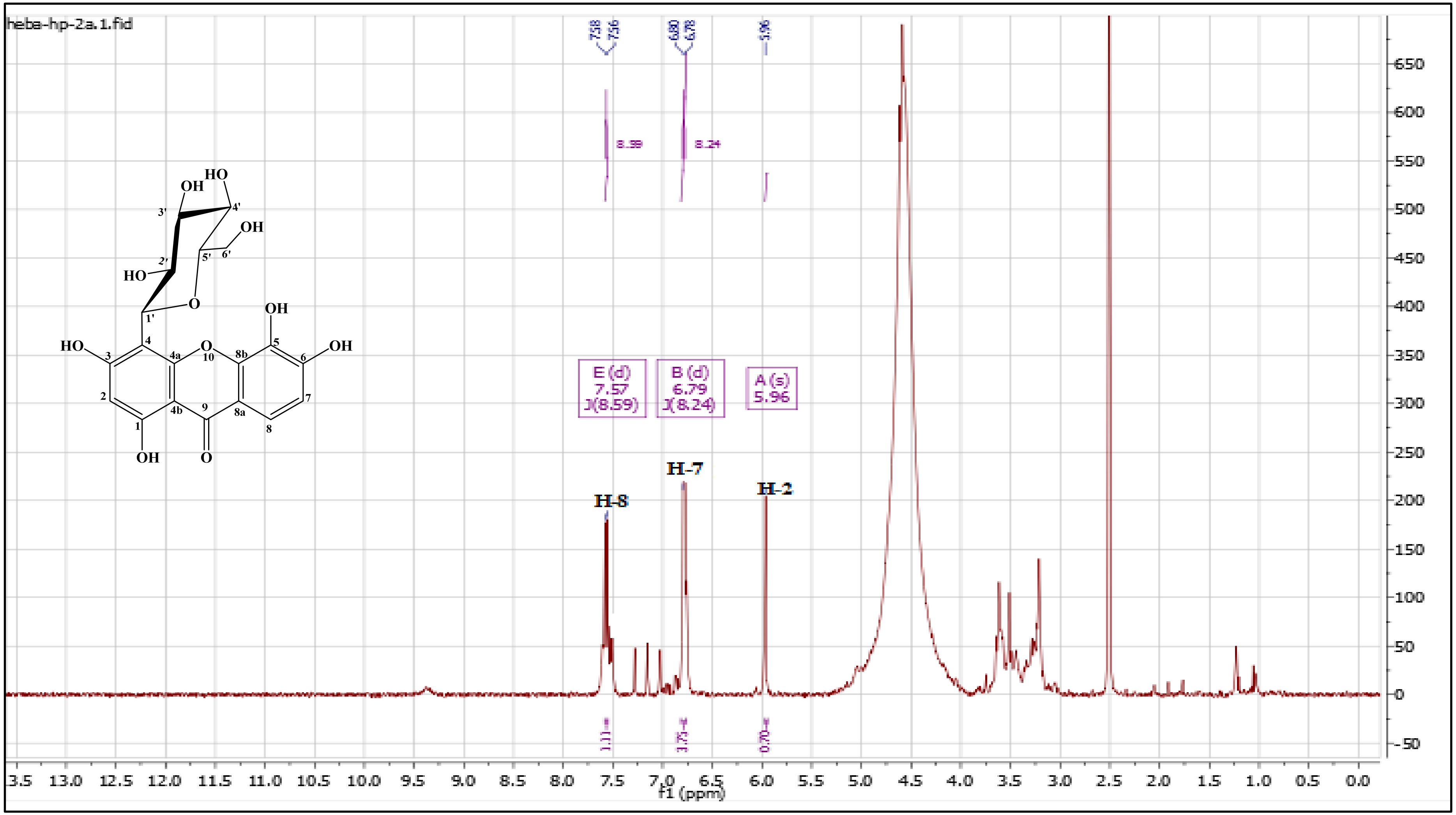

Figure S2. ^{1}H-NMR spectrum of isolated compound (aromatic region).

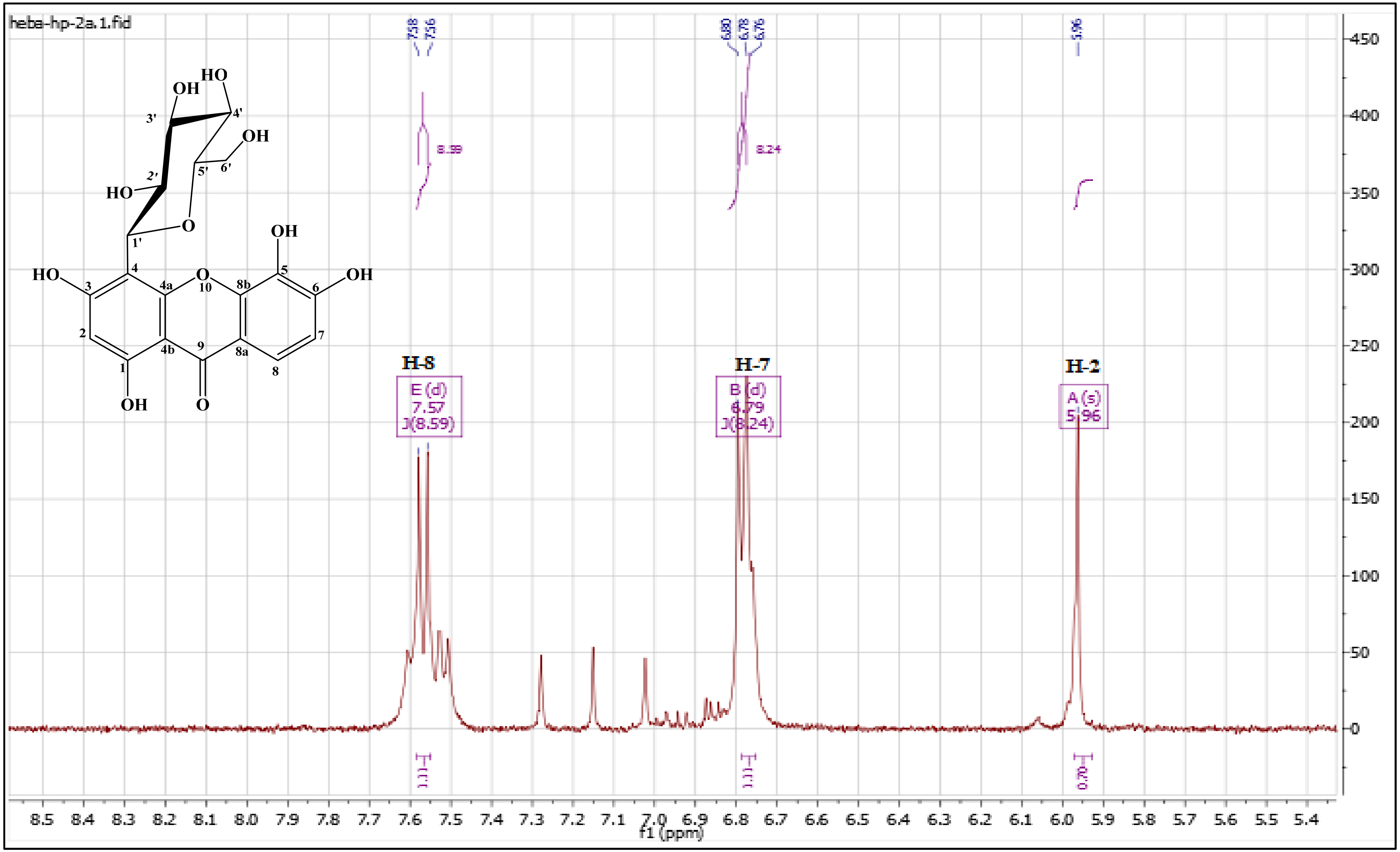

Figure S3. APT spectrum of isolated compound (full spectrum)

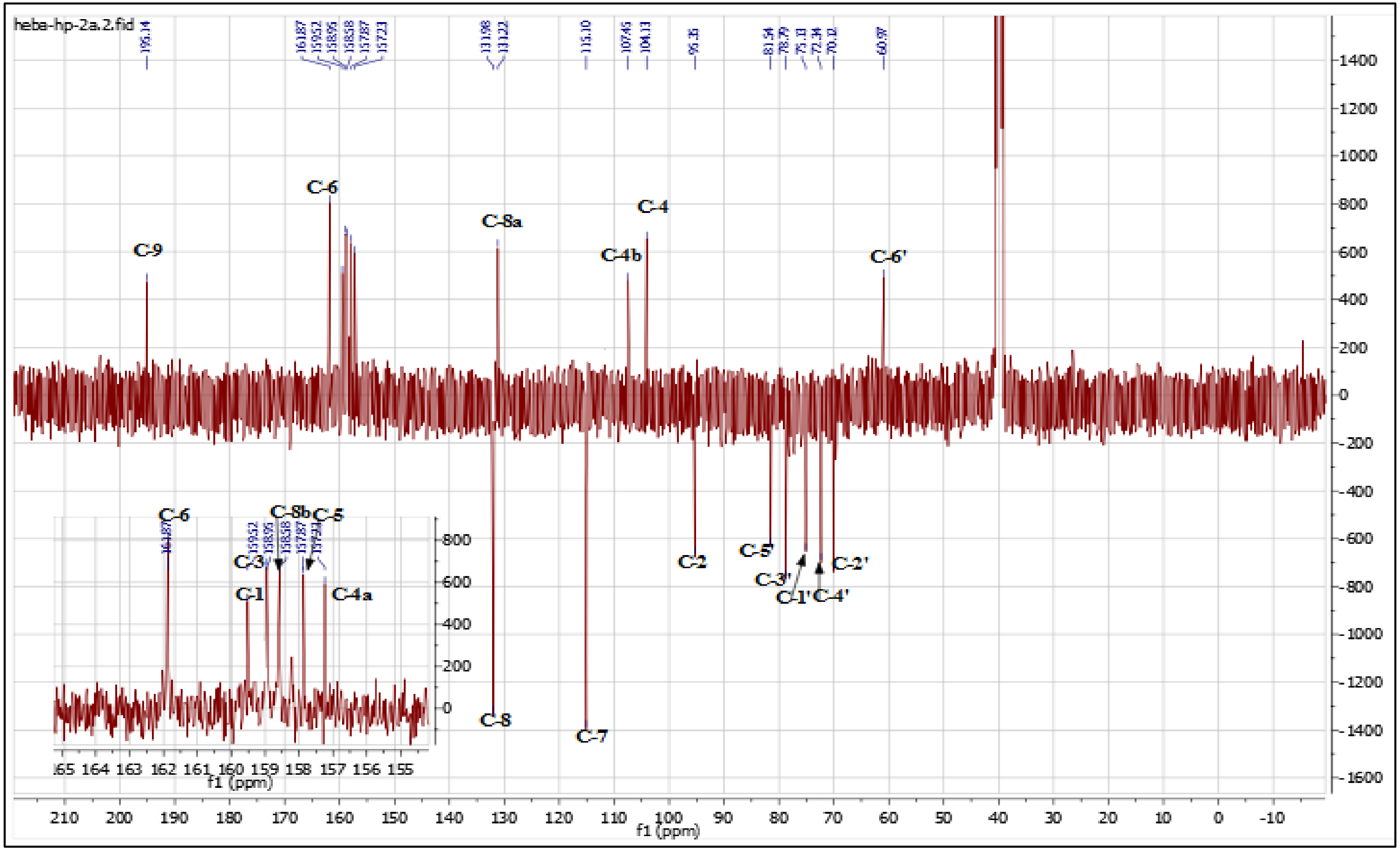

Figure S4. HMBC spectrum of isolated compound

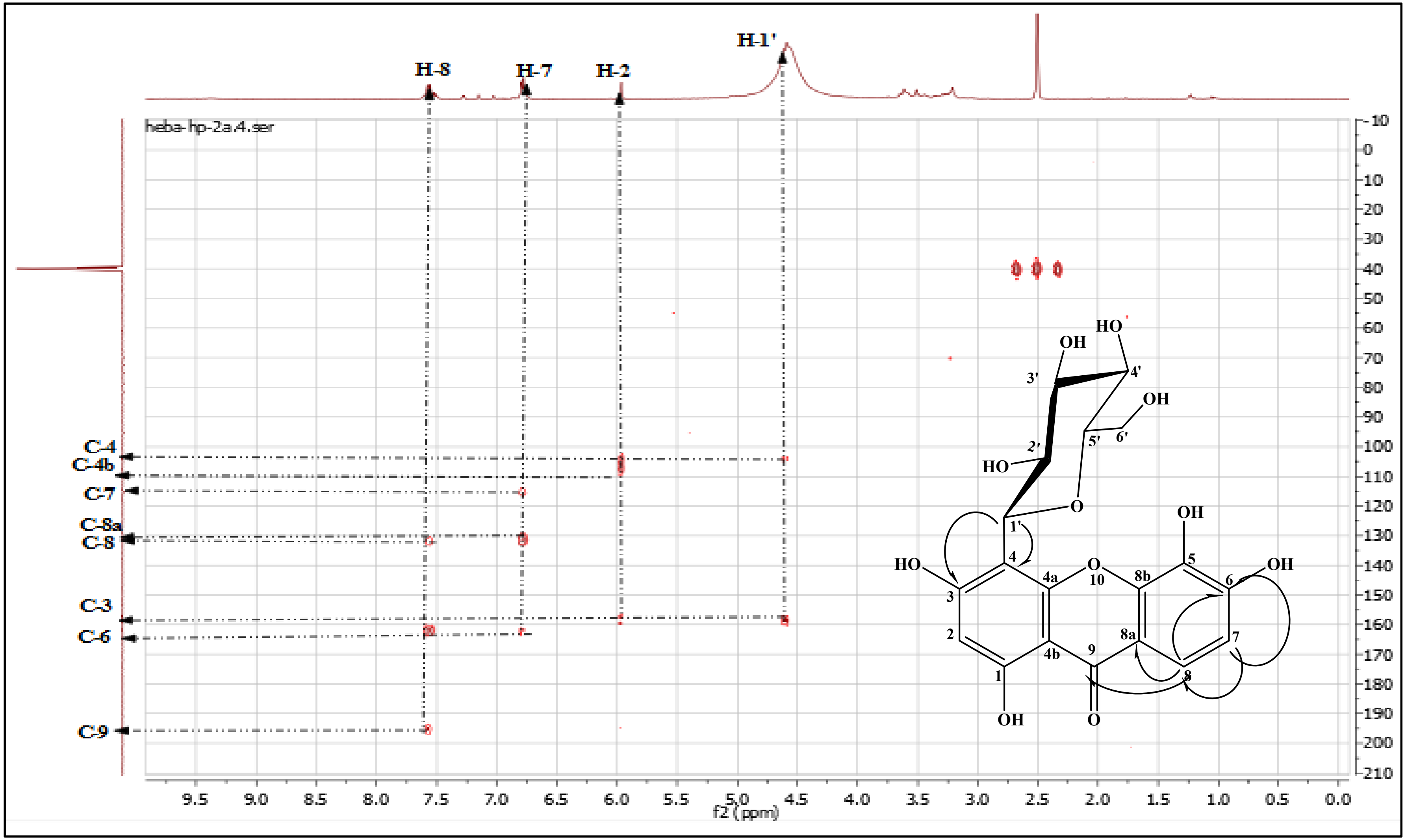

Figure S5. HSQC spectrum of isolated compound

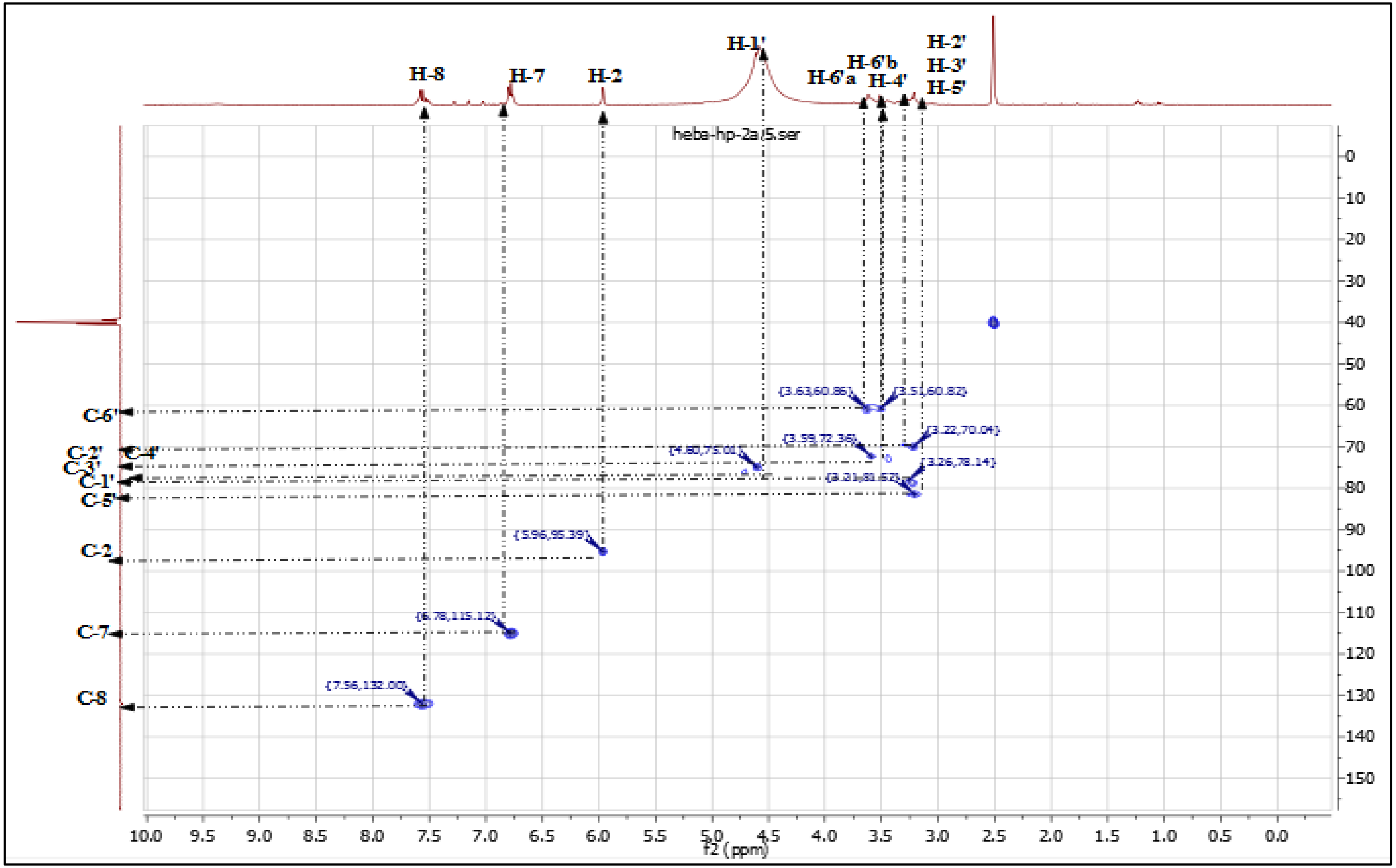

Figure S6. $^{1}H,^{1}H$-COSY spectrum of isolated compound

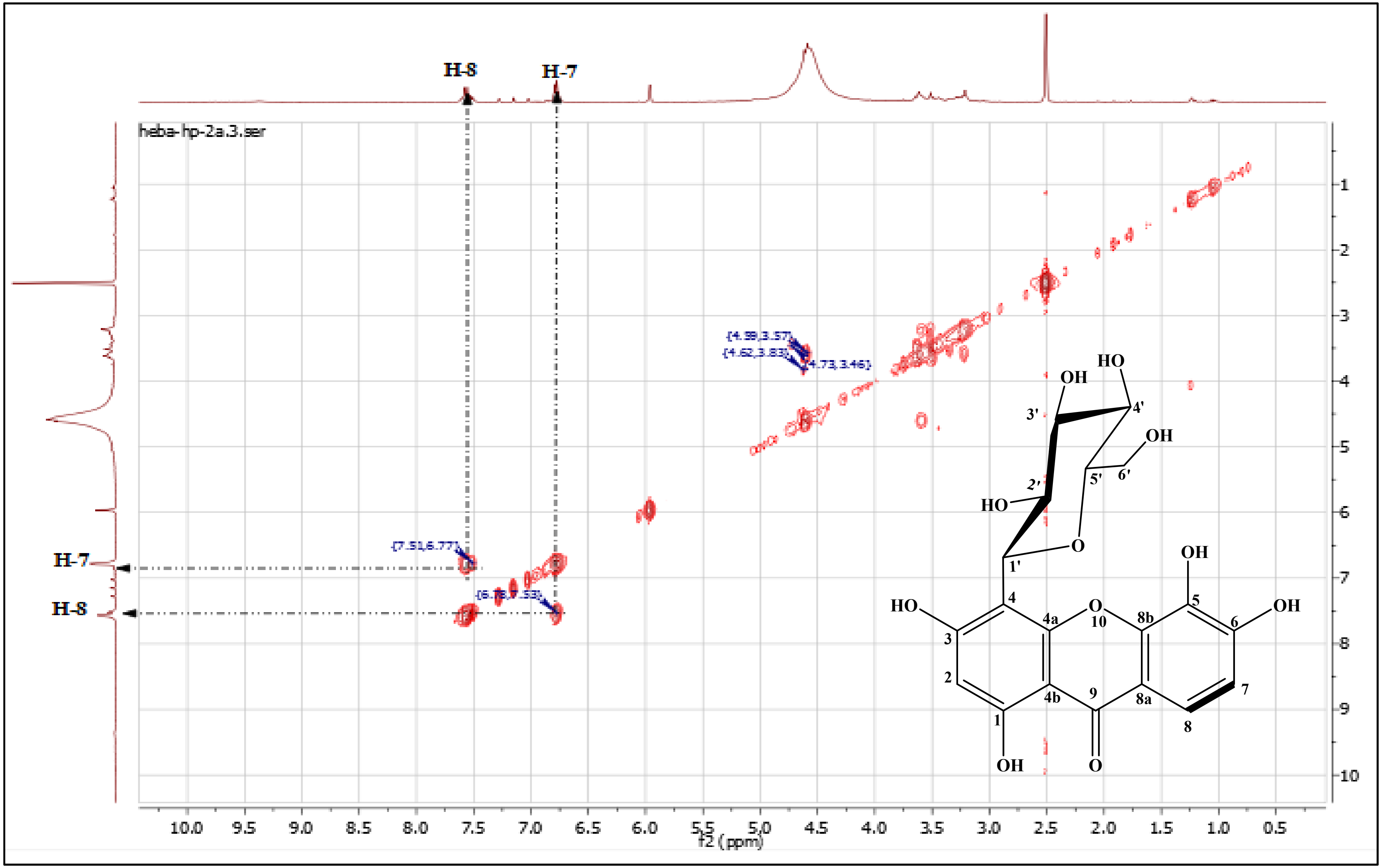

Figure S7. (-)ESI-MS of isolated compound

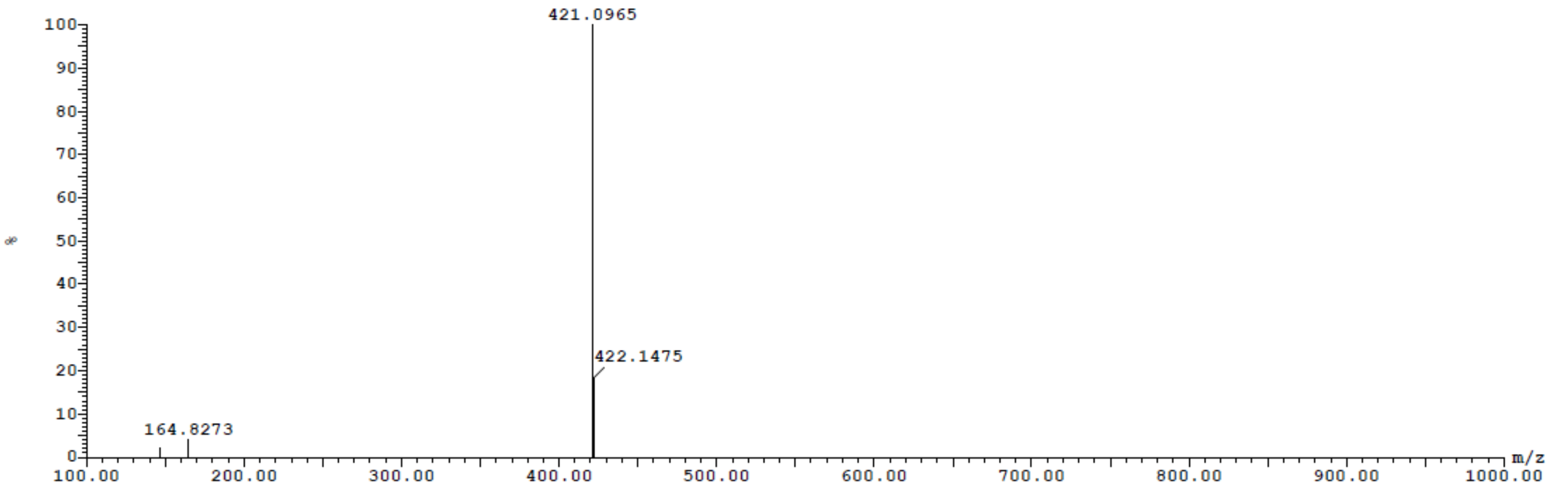

Supplement: Supplementary file 1 [file molecules-27-02609-s001.zip › molecules-1667265-SI.pdf]
